# Sulfasalazine attenuates evading anticancer response of CD133-positive hepatocellular carcinoma cells

**DOI:** 10.1186/s13046-017-0511-7

**Published:** 2017-03-03

**Authors:** Yeonhwa Song, Jaewoo Jang, Tae-Hoon Shin, Sang Mun Bae, Jin-sun Kim, Kang Mo Kim, Seung-Jae Myung, Eun Kyung Choi, Haeng Ran Seo

**Affiliations:** 1 Cancer Biology Research Laboratory, Institut Pasteur Korea, 16, Daewangpangyo-ro 712 beon-gil, Bundang-gu, Seongnam-si, Gyeonggi-do 13488 South Korea; 2 Laboratory of Biochemistry, Division of Life Sciences, Korea University, 145, Anam-ro, Seongbuk-gu, Seoul, 02841 South Korea; 3 Department of Medicine, University of Ulsan College of Medicine, 88 Olympic-ro, 43-gil, Songpa-gu, Seoul, 05505 South Korea; 4 Asan Institute for Life Sciences, Asan Medical Center, University of Ulsan College of Medicine, 88 Olympic-ro, 43-gil, Songpa-gu, Seoul, 05505 South Korea; 5 Division of Gastroenterology and Hepatology, ASAN Medical center, Olympic-ro 43-gil, Songpa-gu, Seoul, 05505 South Korea; 6 Department of Gastroenterology, Asan Medical Center, University of Ulsan College of Medicine, 88 Olympic-ro, 43-gil, Songpa-gu, Seoul, 05505 South Korea; 7 Division of Radiation Oncology, ASAN Medical center, Olympic-ro 43-gil, Songpa-gu, Seoul, 05505 South Korea

**Keywords:** Hepatocellular carcinoma (HCC), Cancer stem cells (CSCs), CD133, Reactive oxygen species (ROS), Sulfasalazine

## Abstract

**Background:**

CD133-positive cells in hepatocellular carcinoma (HCC) exhibit cancer stem cell (CSC)-like properties as well as resistance to chemotherapeutic agents and ionizing radiation; however, their function remains unknown. In this paper, we identified a hitherto unknown mechanism to overcome CD133-induced resistance to anticancer therapy.

**Methods:**

We applied an alternative approach to enrich the CD133-positive HCC population by manipulating 3D culture conditions. Defense mechanisms against reactive oxygen species (ROS) in CSC spheroids were evaluated by fluorescence image-based phenotypic screening system. Further, we studied the effect of sulfasalazine on ROS defense system and synergistic therapeutic efficacy of anticancer therapies both in culture and in vivo HCC xenograft mouse model.

**Results:**

Here, we found that oxidative stress increase CD133 expression in HCC and increased CD133 expression enhanced the capacity of the defense system against ROS, and thereby play a central role in resistance to liver cancer therapy. Moreover, ablation of CD133 attenuated not only the capacity for defense against ROS, but also chemoresistance, in HCC through decreasing glutathione (GSH) levels in vitro. Sulfasalazine, a potent xCT inhibitor that plays an important role in maintaining GSH levels, impaired the ROS defense system and increased the therapeutic efficacy of anticancer therapies in CD133-positive HCC but not CD133-negative HCC in vivo and in vitro.

**Conclusion:**

These results strongly indicate functional roles for CD133 in ROS defense and in evading anticancer therapies in HCC, and suggest that sulfasalazine, administered in combination with conventional chemotherapy, might be an effective strategy against CD133-positive HCC cells.

**Electronic supplementary material:**

The online version of this article (doi:10.1186/s13046-017-0511-7) contains supplementary material, which is available to authorized users.

## Background

Hepatocellular carcinoma (HCC) is the sixth most common malignant tumor, and the second leading cause of cancer-related deaths in the world [1]. Over the past decade, advances in treatment, medical device development, surgical techniques, radiology, and liver transplantation have resulted in considerable improvements in therapy for HCC [2, 3]. However, the prognosis for this disease is still very poor, because most types of HCC are resistant to conventional chemotherapeutic agents and have high recurrence after resection with curative aim. Currently, the only chemotherapeutic agent for HCC is sorafenib, however, it just expands the survival only 2.8 months [4]. Therefore, the novel target and agents for HCC is unmet need.

Cancer stem cells (CSCs) are identified by experiments in which tumor cells are fractionated, characterized by cell surface markers, and injected at limiting dilutions into mice. Those populations that lead to tumor growth in the animal, and that lead to tumor growth when that tumor is subsequently transplanted into a second animal, are considered as CSCs [5]. Studies have shown that CSCs can be resistant to common forms of cancer treatment such as chemo- and radiation therapy, resulting in tumor recurrence, metastasis, and treatment failure [6–8]. Therefore, deeper knowledge of the interactions between cancer cells and CSCs are needed to fully understand tumor development, progression, and chemo-resistance in HCC. Recently, compelling evidence has reported that HCC is hierarchically organized and originates from a primitive stem/progenitor [9].

In particular, CD133 has drawn significant attention as an important liver CSC marker. CD133 was the first identified member of the prominin family of pentaspan transmembrane (5-transmembrane) glycoproteins. It is also commonly known in humans and rodents as Prominin 1 (PROM1) [10]. In HCC, CD133-positive cells exhibit liver CSC-like properties, such as high clonogenicity, tumorigenicity, and resistance to radiation [11, 12]*.* Other studies have shown that the presence of CD133-positive cells in HCC patients after surgery is correlated with early recurrence and poor prognosis [13, 14]*.* However, despite of extensive research efforts, the specific signaling pathway and mechanism of action by which CD133-positive cells are able to evade conventional therapies in HCC or other cancer types remain largely unknown.

Reactive oxygen species (ROS), which are formed by the capture of electrons by an oxygen atom, are chemically reactive molecules that have essential functions in living organisms [15]. In normal cells, moderate levels of ROS are essential for cellular proliferation, differentiation, and survival [16, 17]. On the other hand, chronically increased endogenous ROS levels lead to adaptive changes that play pivotal roles in tumorigenesis, metastasis, and drug resistance in diverse types of cancer cells. Some anti-cancer drugs that increase ROS generation or inhibit ROS elimination can induce a significant accumulation of ROS in cancer cells, leading to oxidative damage and cell death [18]. In recent times, the regulation of ROS levels in CSCs has emerged as an active field of research. CSCs have lower levels of intracellular ROS than do non-CSCs, possibly due to the increased expression of free radical scavenging systems [19–21]. Studies have showed that specific molecules associated with CSCs negatively regulate ROS levels, with a resultant increase in stemness. CD44 is one such molecule that has been associated with CSCs in several types of tumors, promotes ROS resistance by interacting with and stabilizing the cystine/glutamate transporter xCT in human gastrointestinal cancer, and increased CD13 expression reduces ROS levels, promoting the survival of liver cancer stem cells via an epithelial-mesenchymal transition-like phenomenon [22, 23]. However, the roles of CD133 in ROS regulation have not been reported.

In this paper, we show that CD133-positive HCC cells exhibit strong resistance to reactive oxygen species (ROS) via upregulation of glutathione (GSH) levels, and thereby play a central role in resistance to liver cancer therapy. Based on this functional roles of CD133, we also found that sulfasalazine specially modulates the redox status in CD133-positive HCC, and could thereby sensitize CD133-positive HCC to chemotherapeutic treatment. Our results suggest that the combination of sulfasalazine and conventional chemotherapy could potentially be an effective therapeutic strategy against CD133-positive HCC.

## Methods

### Cell culture

Huh7, Hep3B, PLC/PRF/5 and HepG2 cells (human HCC lines) were obtained from the Korean Cell Line Bank. Human HCC cell line Huh6 was kindly provided by Dr. Ralf Bartenschlager (University of Heidelberg, Germany) and Fa2N-4 cells (human immortalized hepatocyte cell line) were purchased from Xenotech (Lenexa, KS, USA). HCC cell lines were cultured in Dulbecco’s minimal essential medium (DMEM; Welgene, Korea, LM001-05) supplemented with heat-inactivated 10% fetal bovine serum (FBS; Gibco, Gaitherburg, MD, USA) and 100U/ml Penicillin and 100 μg/ml Streptomycin (Gibco) at humidified 37 °C incubator under 5% CO_2_. Fa2N-4 cells were plated in collagen-coated plates. After cell attachment (approximately 3 ~ 6 h), serum-containing plating medium (XenoTech, K4000) was replaced with supporting culture medium (XenoTech, K4100.X).

### Primary cell culture

HCC tissue was cut into 3-mm^3^ pieces and washed with 4 °C Hank’s balanced salt solution (Lonza, Basel, Switzerland) supplemented with 1× antibiotic-antimycotic (A/A) solution (Sigma-Aldrich, St Louis, MO, USA) and 1× penicillin-streptomycin (P/S) (Lonza) in 100-mm Petri dishes. After three washes with DMEM/nutrient mixture F-12 (DMEM/F12; Gibco) supplemented with 10% FBS, 1× A/A solution, and 1× P/S, the cells were resuspended in 10 ml of the same solution and incubated at 4 °C for 16 h. Next, tissue was washed with fresh DMEM/F12 and incubated with 2 ml of 2× collagenase II (BD Biosciences, Franklin Lakes, NJ, USA) at 37 °C in a shaking chamber for 90 min. After incubation, the tissues were washed with DMEM/F12 several times until the supernatant was clear. The pellet was resuspended in hepatocyte basal medium (Lonza, CC3199) containing 1× A/A solution, 10% FBS, and 5 μg/ml hepatocyte growth factor (R&D systems, Minneapolis, MN, USA) and plated on collagen type I-coated T-25 flasks (BD Biosciences) with 5×10^5^cells.

### Formation of liver cancer stem cell (LCSC) spheroids

Huh7 were seeded in a very low density on 100-mm dish (5x10^5^cells/10 cm^2^). After attachment of cells, complete medium were changed to DMEM/F12 (Gibco, 10565–018) supplemented with 1 × B27 (Invitrogen, Eugene, OR, USA), 20 ng/ml basic fibroblast growth factor (Invitrogen), 20 ng/ml epidermal growth factor (EGF, Invitrogen), 25 μg/ml insulin (Sigma) (LCSC media). After cultivation for 7 ~ 10 days without changing the medium, floating spheroids were collected and moved to low-attach 6-well plate (Corning, NY, USA) for subculture or 384-well culture plate (Greiner Bio-one, Monroe, NC, USA) for immunostaining.

### Formation of HCC spheroids

For the HCC spheroids culture, slowly pipette the 8 μl of Matrigel (BD bioscience) directly on surface, carefully spread to avoid bubbles, in 384-well culture plates and incubated at 37 °C until Matrigel was solidified. Trypsinized cells were centrifuged at 1200 rpm and resuspended in culture medium and plated onto the Matrigel coated plates at a density of 2 × 10^3^cells/well. Cells were incubated for 30 min at 37 °C to settle to the Matrigel and slowly added 10% Matrigel-medium to the each wells. After maintaining for 5 days, Matrigel-medium was replaced every 2 days. For immunostaining, they were washed with 1 mM Glycine (Sigma) carefully, and then spheroid were moved to 384-well culture plate.

### Detection of drug sensitivity in spheroids

For the drug sensitivity with or without pretreating of sulfasalazine in spheroids, LCSC spheroids and HCC spheroids were transferred to 96-well plate. Spheroid were treated with anti-cancer drug for 6 ~ 8 days, and spheroids were examined their size using Operetta® High Content Screening (HCS) System using × 10 objective in bright field. For SASP pretreating study, spheroid was treated with 200 μM sulfasalazine for 24 h before anti-cancer drug treatment.

### Reagent and antibodies

Hoechst 33342 (H3570, 1:500), 2′,7′-dichlorodihydrofluorescein diacetate, acetyl ester (CM-H_2_DCFDA. C6827, 1:1000), ThiolTracker™ violet (T10095, 1:500), Alexa Fluor® 633 Phalloidin (A22284, 1:100), Goat anti-mouse Alexa Fluor® 488 (A11001, 1:500), Goat anti-rabbit Alexa Fluor® 633 (A21070, 1:500), Goat anti-mouse Alexa Fluor® 633 (A21050, 1:500) and Goat anti-rabbit Alexa Fluor® 488 (A11008, 1:500) were purchased from Molecular Probes (Invitrogen). Rabbit monoclonal anti-CD44 (EPR10133Y clone, ab51037, 1:1000) rabbit polyclonal anti-EpCAM (ab71916, 1:1000) and mouse monoclonal anti-CD90 (ab133350, 1:1000) were purchased from Abcam (CSP, Cambridge, England). Methotrexate, Doxorubicin, Cisplatin, Sorafenib, Sulfasalazine (SASP), Buthionine sulphoximine (BSO), Arsenic trioxide, Etoposide and Hydrogen peroxide (H_2_O_2_) were purchased from Sigma Chemicals. Mouse monoclonal anti-CD133/1 (AC133, 130-090-422, 1:100) was purchased from Miltenyi biotec (Bergisch Gladbach, Germany). Rabbit polyclonal anti-AFP (Dako, Denmark A/S, Denmark, A000829. 1:500) and mouse monoclonal anti-β-actin (Sigma, A5441, 1:10,000) antibodies were purchased from each of the indicated companies.

### HCS imaging assay technology

Cells were seeded at a 2,500 cells/well density for 72 h incubation and 1,500cells/well density for 96 h incubation in 384-well plate (90% of confluence in analyzing day). After being treated with the indicated concentrations of various drugs for proper time, cells were washed with Dulbecco’s phosphate buffered saline (DPBS; Welgene) and stained with fluorescent probes or antibody. Automated live-cell multispectral image acquisition was performed on the Operetta® HCS System using × 20 objective (Perkin Elmer, Waltham, MA, USA). The fluorescence images were captured according to the optimal excitation and emission wavelengths of each probe. To capture enough cells (>100) for analysis, five image fields starting at the center of well were collected from each well. Image analysis was performed using the Image Mining software. A series of measurements from the nuclei, ROS, and ThiolTracker™ channel images were obtained for each drugs.

### siRNA transfection

siRNA probes were designed by and purchased from Dharmacon (Lafayette, CO, USA). Huh7 cells were seeded with 1x10^6^cells/10 cm^2^ and the medium was replaced with Opti-MEM (Gibco) when the cell density reached 40–50%. The sequences of siCD133 was as follows: CD133 #1, 5′-GCUAAGUACUAUCGUCGAA-3′; CD133 #2, 5′-GAACAAGUUUACAGUGACU-3′; CD133 #3, 5′-GAAGUAUGGGAGAACAAUA-3′; CD133 #4, 5′-UCACAAUCCUGUUAUGACA-3′; Cells were co-transfected with the four siRNAs targeting CD133 (siCD133) scramble (siCont) for 24 h using Lipofectamine® 2000 (Invitrogen).

### Cell sorting

Huh7 cells were analyzed by fluorescence-activating cell sorting (FACS; BD bioscience). The cells were harvested using 0.05% trypsin (Gibco), washed twice with DPBS supplemented with 5% FBS and resuspended in DPBS supplemented with 10% FBS with mouse anti-human CD133/1 antibody (Miltenyi Biotec) for 30 min at 4 °C. The cells were washed twice with pre-cooled DPBS and centrifuged at 1200 rpm at 4 °C and incubated in DPBS supplemented with 10% FBS with goat anti-mouse Alexa Fluor® 488 for 30 min at 4 °C in dark. After washing with DPBS twice, cells were sorted by FACS. CD133^−^negative and CD133-positive HCC were collected for further experiments, and they were cultured in DMEM supplemented with 10% FBS with 1% of penicillin/streptomycin.

### Flow cytometry analysis

For investigating the CD133 population after CD133 positive cell sorting or transfected with siCD133, cells were trypsinized and washed twice with DPBS supplemented with 5% FBS and resuspended in DPBS supplemented with 10% FBS with mouse anti-human CD133/1 antibody for 30 min at 4 °C. The cells were washed with DPBS and centrifuged at 1200 rpm a 4 °C and incubated n DPBS supplemented with 10% FBS with goat anti-mouse Alexa Fluor® 488 for 30 min at 4 °C in dark. After washing with DPBS twice, cells were analyzed by flow cytometry.

For analyzing of cellular ROS levels, the cells were incubated with 10 mM of CM-H_2_DCFDA at 37 °C for 10 min in the dark, and washed with DPBS. For detecting the GSH levels, the cells were washed with DPBS containing the Ca^2+^ and Mg^2+^ and then cells were treated with 20 mM ThiolTracker™ in DBPS containing the Ca^2+^ and Mg^2+^ at 37 °C incubator for 30 min in the dark. Washed cells were then trypsinized and suspended for flow cytometry, intensity of 405 nm were measured for detecting GSH level.

### Analysis of total glutathione (GSH) activity

Assay for total GSH activity was performed using assay kit (Sigma) according to the manufacturer’s protocol. The method is based on the 5,5′-dithiobis-(2-nitrobenzoic acid) (DTNB) reaction and the products were read at 412 nm.

### Polyacrylamide gel electrophoresis (PAGE) and western blot analysis

Cells were solubilized in lysis buffer (3 M, Maplewood, MN, USA), the samples were boiled for 5 min, and equal amounts of protein (10–30 μg/well) were separated on 8 or 10% SDS-PAGE gels. After electrophoresis, the proteins were transferred onto a polyvinylidene difluoride (PVDF) membrane (Millipore, Billerica, MA, USA) and blocked with 5% skim milk for 30 min at R.T. After blocking, the PVDF membranes were incubated with anti-CD133, xCT and β-actin for 16 h at 4 °C. After washing, the blots were incubated with horseradish peroxidase-conjugated secondary antibody (Cell Signaling Technology, Danvers, MA, USA) at a 1:10000 dilution, and specific bands were visualized by enhanced chemiluminescence (ECL; Thermo Scientific) and recorded on X-Omat AR films (Eastman Kodak Co., Rochester, NY, USA).

### Irradiation

Cells were plated in 100-mm dish and 6-well plate for each experiment, and tumor-bared mice were treated with 1Gy, 2Gy, 4Gy (for colony forming assay), 5Gy (xenografted mice), 10 Gy (cell survival, ROS accumulation) of ioning radiation (IR) using a 6 MV photon beam linear accelerator (CL/1800, Varian Medical System Inc., Palo Alto, CA, USA) [24, 25].

### Clonogenic survival assays

Briefly, Huh7-siCont and Huh7-siCD133 cells were seeded into 6-well plates (Nunc, Roskilde, Denmark) at a density of 500 cells/well and allowed to grow for 24 h. The cells were then treated with SASP (100 μM or 200 μM) for further 24 h [26]. The SASP-containing media were then discarded, and the cells were washed with DPBS and culture to form colonies in complete medium after irradiation (1, 2, 4Gy).

### Tumor xenografts in nude mice

Huh7 (5x10^6^cells) with 95% viability were injected subcutaneously into the hind legs of 6-week-old BALB/c athymic nude mice (SLC Inc., Hamamatsu, Japan) [27, 28]. When tumors reached a volume of 200–250 mm^3^, mice were randomly allocated to four groups as follows: (1) the tumor control group, (2) the SASP group (5 mg/20 g, 9 days) [22], (3) the IR group (5Gy), and (4) the SASP plus IR group. Each group contained three mice. Tumor volumes were determined using the following formula: (L × I^2^)/2, where L = tumor length and I = tumor width. Dimensions were determined using calipers. Local tumor irradiation was performed under anesthesia using a 6 MV photon beam linear accelerator (CL/1800). SASP was dissolved in saline (0.9% NaCl) and injected for intraperitoneal for 9 days. The mice were sacrificed for immunohistochemistry, RT-PCR and western blot after 15 days from SASP injection.

### Statistical analysis

All experiments were performed at least three times. The results are expressed as the mean ± SD. Statistical analysis was performed using the Student’s *t*-test.

## Results

### CD133-positive cells have cancer stem cell-like properties in liver cancer

**Fig. 1:**
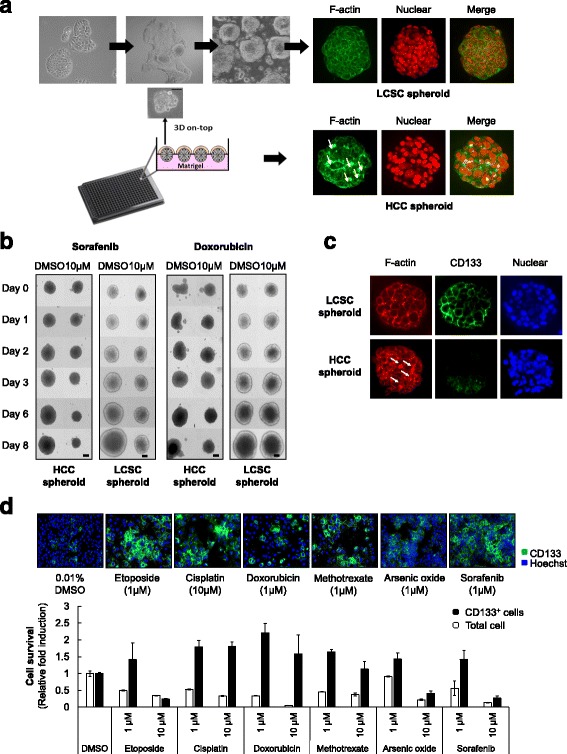
The CD133-positive HCC population has cancer stem cell-like properties in liver cancer. **a** Schematic of the experimental procedure for the generation of LCSC and HCC spheroids (*left panel*). Spheroids were stained with phalloidin-633, which is staining the F-actin for structure morphology, and Hoechst 33342 for nucleus staining in order to compare the different morphological signatures between the two spheroids. **b** The drug sensitivities of LCSC and HCC spheroids were examined after treatment with 10 μM sorafenib and doxorubicin for 8 days. The size of the spheroid was measured each day using the Operetta® High Content Screening System using a × 10 objective (scale bar: 200 μm). **c** LCSC and HCC spheroids were stained with CD133 and phalloidin-633, which identified CSCs in both spheroids. **d** Chemoresistance of the CD133-positive HCC population to anti-cancer drugs was detected using the HCS system. CD133 staining was carried out following drug treatment for 48 h (*upper panel*); mean values ± SD from two independent experiments for cell survival ratio relative to the value for control are shown in the *lower panel*

In order to overcome resistance to chemotherapy in cancer stem cells (CSCs), these CSCs should be characterized in particular cancer types. To identify CSC markers specific to HCC, we generated HCC spheroid and liver cancer stem cell (LCSC) spheroid from the Huh7 cell line in different culture conditions to overcome the shortcomings of the monolayer culture system in vitro. Additionally, Huh7 is the best proper human HCC cell lines for high content screening, cell sorting with CD133 antibody, and xenograft mouse model, therefore, we used Huh7 cell lines as representative human HCC cells. LCSC spheroids were grown in serum-free medium supplemented with growth factors. Only floating spheroids were collected as the LCSC population (Fig. 1a, upper). On the other hand, HCC spheroids were generated on the Matrigel-coated plates in DMEM with 10% fetal bovine serum (FBS) (Fig. 1a, lower). In order to analyze the differences between spheroids grown in different environments, we compared various characteristics, such as functional structure, drug sensitivity, and composition, between the LCSC and HCC spheroids.

Since bile canaliculi, which play an important role in maintaining liver function, contain many F-actin microfilaments, we compared the F-actin pattern to fine different morphological signatures between LCSC and HCC spheroids. F-actin staining was concentrated into bile canaliculi-like structures (indicated by white arrows in Fig. 1a) in HCC spheroids, while F-actin merely localized to cell membranes in LCSC spheroids (Fig. 1a). Moreover, LCSC spheroids express a lower level of hepatocyte-related mRNA (albumin, HNF1a) and higher level of stem cell-related mRNA (OCT4 and TERT) compared to HCC spheroids (Additional file 1: Figure S1). Thus, we speculated that LCSC spheroids could not perform the main functions of liver cells, since they did not exhibit similar architecture to spheroids derived from neoplastic liver tissue, compared to HCC spheroids.

Next, we investigated cell viability in both types of spheroids following exposure to common anti-cancer drugs against HCC (Fig. 1b). Following treatment with sorafenib and doxorubicin, we observed a significant decrease in the size of HCC spheroids (sorafenib: 421 μm to 312 μm, doxorubicin: 471 μm to 342 μm) whereas the average size of the LCSC spheroids remained the same or continuously increased (sorafenib: 332 μm to 487 μm, doxorubicin: 418 μm to 672 μm). We obtained similar results with other anticancer drugs such as taxol and etoposide (Additional file 1: Figure S2). These results demonstrate that LCSC spheroids have strong resistance to chemotherapy compared to HCC spheroids. Moreover, these results suggest that the tumor microenvironment plays a critical role in modulating drug sensitivity, because differences in tumor growth environments resulted in different sensitivities to chemotherapy, even though these spheroids were formed from same HCC cell line.

To determine the underlying cause of the increased drug resistance in LCSC spheroids, we investigated the expression of CSC-associated cell surface markers (CD133, EpCAM, CD90, CD44) in LCSC spheroids. To present the spheroid staining image, we set the Z’ stack on spheroid using the high content screening system. It means that every 10 μm images on spheroid were taken and merged together. CD133 staining in HCC spheroid is uneven, on the other hand, expression of CD133 in LCSC spheroid was much even compared to HCC spheroid. LCSC spheroids contained a large population of CD133-positive cells, whereas HCC spheroids included only a small population of CD133-positive cells, as these HCC spheroids exhibit relatively higher heterogeneity (Fig. 1c). Two kinds of spheroids were derived from the Huh7 cells, however, they showed different F-actin pattern and CD133 expression.

Based on earlier observations that CD133-positive cells exhibit resistance to chemotherapy, we investigated sensitivities to various anticancer drugs in monolayer cultured HCC using the high content screening (HCS) system. In Fig. 1d, we tested the chemoresistance in HCC cell line using cisplatin, doxorubicin, etoposide, methotrexate, As_2_O_3_ and sorafenib. Except sorafenib and cisplatin, other drugs are not commonly used in HCC patients, however, we would like to examine the ROS inducing drugs in cancer stem cells. Indeed, the CD133-positive cell population exhibited strong viability in the presence of anti-cancer drugs in contrast to a decrease in total cell number in HCC (Fig. 1d). These results indicated that LCSC spheroids and HCC spheroids have different composition and drug sensitivities, although both spheroids originated from the same HCC cell line. Collectively, these results suggest that CD133-positive cells possess cancer stem cell-like characters in liver cancer.

### Oxidative stress-induced CD133 overexpression facilitates an efficient ROS defense system

**Fig. 2:**
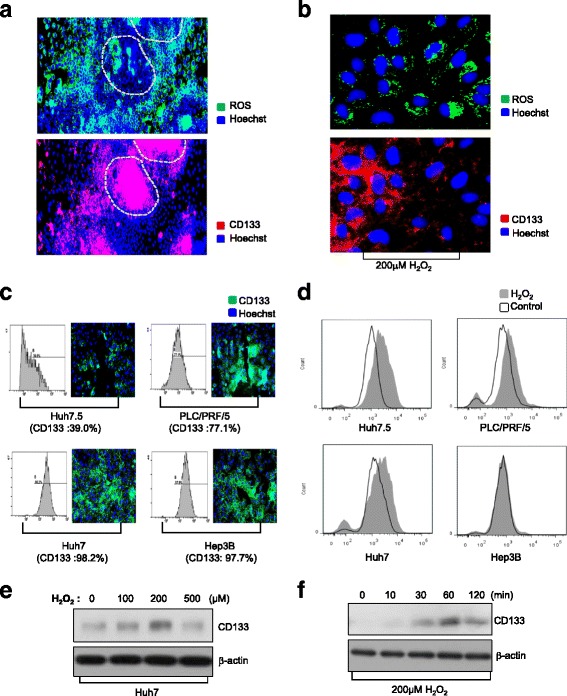
Overexpression of CD133 inhibits ROS accumulation. **a** LCSC spheroids were reattached in a 384-well culture plate and stained with CM-H_2_DCFDA and CD133 to investigate the relationship between CD133 expression and intracellular ROS levels. **b** Expression of CD133 and ROS accumulation were detected after treatment with 200 μM H_2_O_2_ for 20 min in 2D culture condition. **c** Expression of CD133 in HCC cell lines was measured using flow cytometry and immunostaining analysis. **d** HCC cell lines were treated with 200 μM H_2_O_2_ for 20 min. ROS accumulation was analyzed by flow cytometry. **e-f** Huh7 cells were treated with the indicated concentrations of H_2_O_2_ and time. Whole cell lysates were then prepared and assessed by western blotting. Expression of CD133 was examined depend on the concentration (**e**) and treatment time (**f**) of H_2_O_2_. ß-actin was used as control

In order to overcome CD133-induced chemoresistance, we focused on elucidating the functional roles of CD133 in chemoresistance. To define detailed mechanisms of drug resistance in the CD133-positive cell population, we investigated differences between CD133-positive and CD133-negative HCC populations by utilizing LCSC spheroids. Following the generation of LCSC spheroids, we modified their environment from the stem cell-permissive medium (DMEM/F12 supplemented 1× B27, 20 ng/ml bFGF, 20 ng/ml EGF, 25ug/ml insulin) to a general HCC culture medium (DMEM-high glucose supplemented with 10% FBS) to make CD133-negative HCC populations, which were differentiated from LCSC spheroids. After 2 days, we observed that LCSC spheroids were tightly attached on the bottom of the culture dish. Under these conditions, we investigated the pattern of reactive oxygen species (ROS) accumulation in live conditions by fluorescence microscopy after staining with CM-H_2_DCFDA, since ROS are generally known as mediators of apoptosis induced by various anti-cancer drugs. ROS were noticeably detected in the outer surface cells of the LCSC spheroids, whereas relatively small amounts of ROS were seen in the center of the LCSC spheroids (Fig. 2a, *upper panel*).

Next, we investigated the distribution of CD133 expression at the same positions. The center of the LCSC spheroid maintained high CD133 expression, whereas the outer surface cells of the LCSC spheroid quickly lost CD133 expression (Fig. 2a, *lower panel*). In terms of the macroscopic trends in ROS accumulation and CD133 expression, ROS content tended to be inversely proportional to CD133 expression. We also detected that cells with high CD133 expression could inhibit ROS production, relative to cells with low CD133 expression, following exposure to oxidative stress (200 mM, H_2_O_2_) in conventional 2D culture conditions (Fig. 2b).

To ascertain the effect of CD133 on ROS regulation, we selected four human HCC lines that exhibit different expression levels of CD133. FACS and immunostaining analysis revealed that different HCC cell lines have different proportions of CD133-positive cells, in the following order: Huh7 (98.2%), Hep3B (97.7%) > PLC/PRF/5 (77.1%) > Huh7.5 (39.0%) (Fig. 2c). When these HCC lines were exposed to H_2_O_2_, remarkably, ROS accumulation was inversely proportional to CD133 expression in the order Huh7.5 > PLC/PRF/5 > Huh7, Hep3B (Fig. 2d).

We next studied whether the expression of CD133 could be modulated by oxidative stress. For this, HCC cells that were exposed to H_2_O_2_ were subjected to CD133 expression analysis. Treatment with H_2_O_2_ significantly increased CD133 expression in a dose-dependent manner until 200 μM (Fig. 2e) and time-dependent manner until 60 min (Fig. 2f) in HCC. These results suggest that the oxidative stress increase CD133 expression and overexpression of CD133 may play a role in ROS defense in HCC.

### Increased CD133 expression reduces intracellular ROS via upregulation of GSH levels and thereby promotes resistance to anti-cancer therapies

**Fig. 3:**
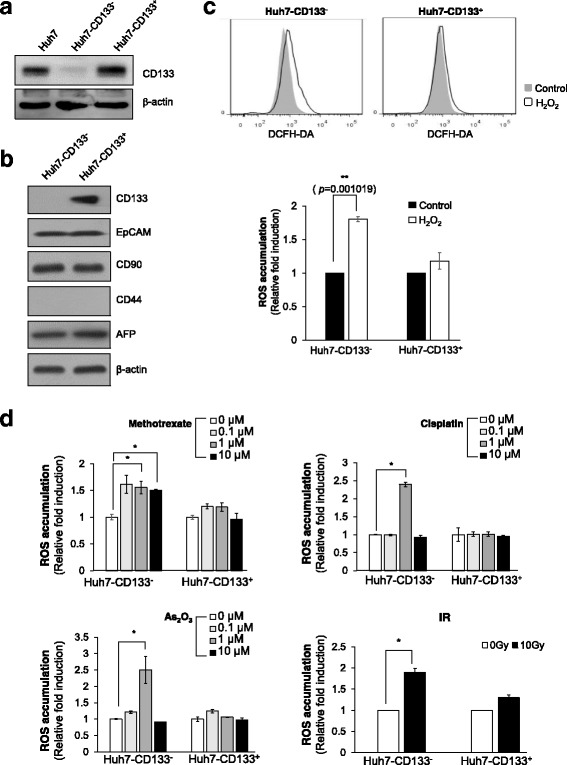
CD133-positive HCC cells have an efficient ROS defense system. **a** Western blot analysis of CD133 after sorting of CD133-negative and CD133-positive HCC from Huh7 cells. **b** Expression of CSC-related cell surface markers (EpCAM, CD90, CD44, CD24, AFP) were examined in CD133-negative HCC and CD133-positive HCC by western blotting. **c** ROS accumulation in CD133-negative and CD133-positive HCC was measured after treatment with 200 μM H_2_O_2_ for 20 min. The data shown were from three independent experiments relative to the value for control. **d** CD133-negative and CD133-positive HCC were treated with the indicated concentrations of MTX, cisplatin, and As_2_O_3_. To examine the effect of radiation, cells were treated with 10 Gy radiation. For ROS level measurement, cells were stained with CM-H_2_DCFDA and analyzed by the HCS system after 12 h treatment with anti-cancer drugs and radiation treatment

To elucidate the functional relevance of CD133 expression to intracellular ROS levels, we sorted CD133-positive and CD133-negative HCC cells from Huh7 cells and then we confirmed sorting efficiency by western blotting against CD133 (Fig. 3a). Western blotting showed that CD133-positive and CD133-negative HCC cells did not exhibit differences in the expression of other CSC-associated cell surface markers, such as EpCAM, CD90, CD44, and AFP and expression of CD44 was hardly detected in huh7 cells (Fig. 3b). These results represented that difference of CD133 expression could not alter the expression of other CSC-associated cell surface markers.

Next, we treated H_2_O_2_ in CD133-positive and CD133-negative HCC cells. ROS production was obviously increased in CD133-negative HCC cells, whereas significant ROS accumulation was not observed in CD133-positive HCC cells following oxidative stress (Fig. 3c). To assess the effects of CD133 on the regulation of ROS accumulation by chemotherapeutic agents and ionizing radiation (IR), we exposed both populations to various ROS inducers such as As_2_O_3_, cisplatin, methotreaxate and IR. CD133-negative HCC cells displayed significant increase in ROS accumulation by ROS inducers, however, CD133-positive HCC cells did not exhibit enhanced ROS accumulation under the same conditions (Fig. 3d).

**Fig. 4:**
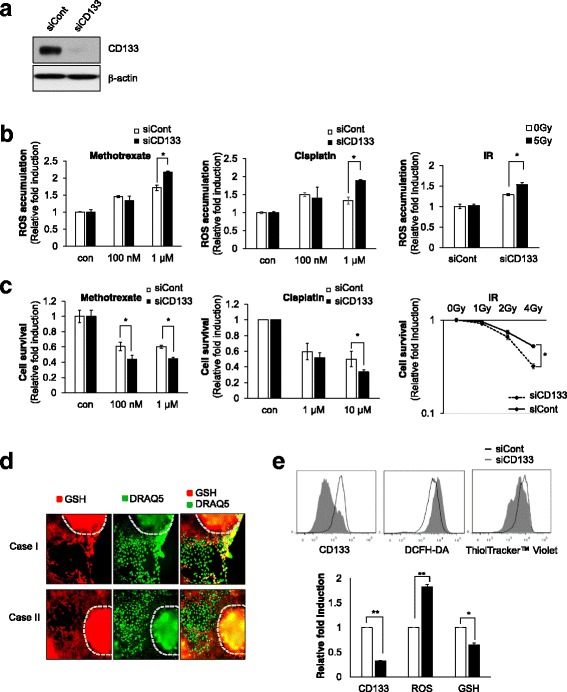
CD133 regulates intercellular ROS via upregulation of GSH levels and leads to resistance to anti-cancer therapies. **a** Huh7 was transfected with siCont and siCD133, and their efficiency was examined by western blot analysis. **b** For the measurement of ROS levels, Huh7-siCont and Huh7-siCD133 cells were treated with the indicated concentrations of MTX and cisplatin for 12 h and 5 Gy of radiation. **c** Huh7-siCont and Huh7-siCD133 cells were treated with the indicated concentrations of MTX and cisplatin for cell survival analysis. After treatment for 48 h, nuclei were stained with Hoechst33342 and counted. For the colony-forming assay, Huh7-siCont and Huh7-siCD133 cells were treated with 1, 2, or 4 Gy of radiation. Cells were allowed to grow for 14 days, stained with trypan blue, and scored. Data represent values ± SD from three independent experiments relative to the value for control. **d** LCSC spheroids were reattached on a 384-well culture plate and GSH levels were determined by ThiolTracker™ Violet staining using the HCS System. **e** CD133 levels, ROS accumulation, and GSH were investigated by flow cytometry in Huh7-siCont and Huh7-siCD133. Each value of Huh7-siCD133 was normalized to Huh7-siCont. *p < 0.05. **p < 0.005

For a more accurate analysis, we also investigated the effects of CD133 ablation on the ROS defense system. SiRNA of CD133 (siCD133) efficiently depleted CD133 protein levels, whereas control siRNA (siCont) treatment did not (Fig. 4a). CD133-deficient cells manifested a greater increase in ROS than did siCont-transfected cells following exposure to ROS inducers (MTX, cisplatin, and IR) (Fig. 4b). Because depletion of CD133 increased ROS accumulation, we next examined whether depletion of CD133 could modulate cell fate. CD133 depletion facilitated chemotherapy or radiation-induced cell death (Fig. 4c). Taken together, these results suggest that CD133 might promote the ROS defense system and thus enhance resistance to chemotherapy and IR treatment in HCC.

Because cancer cells are protected against oxidative stress by an interacting network of antioxidant enzymes, we investigated the expression of antioxidant genes in CD133-depleted Huh7 cells by reverse transcription polymerase chain reaction (RT-PCR) (Additional file 1: Figure S3). However, CD133 depletion did not affect the expression of antioxidant genes. This suggests that CD133-mediated ROS resistance is independent of the regulation of antioxidant gene expression. Among antioxidant molecules, glutathione (GSH) is found in particularly high levels in the liver tissues and serves in detoxification metabolism. Thus, we compared GSH levels between the central cells and outer surface cells of LCSC spheroids like as Fig. 2a. The cellular level of GSH was estimated by staining of ThiolTracker™ Violet, as GSH represents the majority of intracellular free thiols in the cell. As expected, large amounts of GSH presented in the cells inside the LCSC spheroid, relative to the outer surface cells of the LCSC spheroid (Fig. 4d). FACS analysis also clearly revealed that CD133 depletion significantly increased ROS accumulation via inhibition of GSH levels (Fig. 4e). Taken together, these results suggest that CD133 attenuates intracellular ROS accumulation through the enhancement of GSH levels and thereby promotes resistance to anti-cancer therapy in HCC.

### Sulfasalazine enhances the efficiency of anticancer therapies in CD133-positive HCC but not CD133-negative HCC

**Fig. 5:**
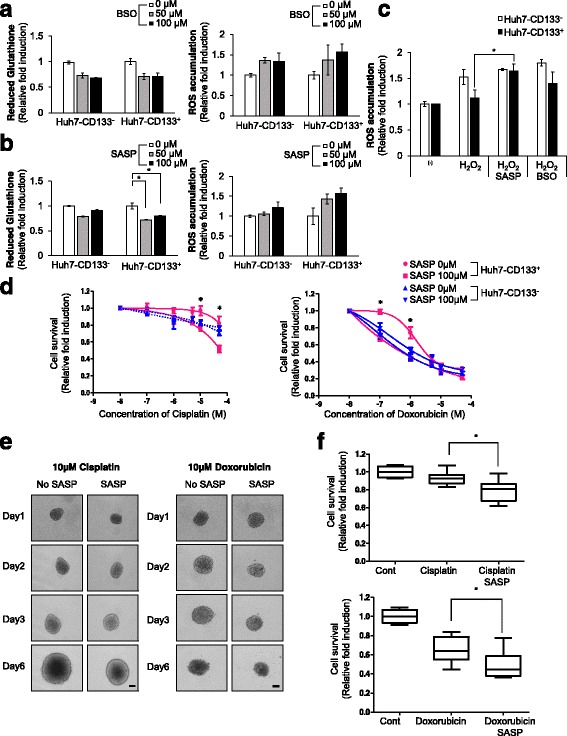
Sulfasalazine (SASP), an inhibitor of xCT, overcomes chemoresistance to anti-cancer therapies in CD133-positive HCC but not in CD133-negative HCC. **a**, **b** ROS accumulation and reduced glutathione were measured by treating with the indicated concentrations of (**a**) buthionine sulphoximine (BSO), and (**b**) sulfasalazine (SASP) for 24 h in CD133-negative and CD133-positive HCC. After treatment, levels of ROS and GSH were examined using the HCS System via staining with CM-H_2_DCFDA and ThiolTracker™ Violet. **c** CD133-negative and CD133-positive HCC were pretreated with SASP or BSO before being treated with 200 μM H_2_O_2_ for 24 h. After treatment, levels of ROS was examined using the HCS System. **d** Dose response curve of CD133-negative and CD133-positive HCC cells, constructed from the results of pretreatment with 200 μM SASP for 24 h before cisplatin, MTX, and sorafenib treatment with indicated concentration for further 48 h. After 48 h of drug treatment, the nucleus was stained with Hoechst33342 and counted (*upper panel*). **e** LCSC spheroids were formed and treated with doxorubicin, cisplatin for 6 days with or without pretreatment with 200 μM SASP. The size of LCSCs was detected with bright field microscopy with a 10 × objective (scale bar: 100 μm). **f** Human primary HCC cells were treated with anti-cancer drugs (cisplatin, doxorubicin) for 48 h, with or without pretreatment with 200 μM SASP for 24 h. Nuclei were stained with Hoechst33342 and examined using the HCS System. All data are mean values ± SD from two independent experiments. *p < 0.05, **p < 0.005

To explore the underlying mechanism by which CD133 contributes to GSH synthesis, we investigated the effects of GSH inhibitors, which deplete GSH by inhibiting its synthesis, in CD133-positive and CD133-negative HCC. Buthionine sulphoximine (BSO) is an inhibitor of gamma-glutamylcysteine synthetase (gamma-GCS), and sulfasalazine (SASP) is a specific inhibitor of xCT-mediated cystine transport. BSO inhibited GSH levels and increased endogenous ROS to a similar extent in both populations (Fig. 5a), whereas SASP treatment resulted in higher GSH inhibition and ROS production in CD133-positive HCC cells than in CD133-negative HCC cells (Fig. 5b). We also examined total GSH activity using assay kit, and they exhibited similar effect as shown in Fig. 5a, b (Additional file 1: Figure S4-A, B). Moreover, the strong ROS defense capacity of CD133 positive cells following H_2_O_2_ exposure was attenuated by pretreatment with SASP but not by pretreatment with BSO (Fig. 5c).

Next, we investigated whether pretreatment with SASP can overcome resistance to anticancer therapy in CD133-positive HCC cells. Here, we pre-treated sulfasalazine 24 h before the anti-cancer treatment, because we investigated the changing levels of reduced GSH and ROS after treatment with sulfasalazine for 24 h with lower concentration than 200 μM in Fig. 5b.

Cell number was measured following treatment with cisplatin, doxorubicin, which can induce ROS accumulation, with or without pretreatment with sulfasalazine in CD133-positive HCC and CD133-negative HCC cells: pretreatment with sulfasalazine dramatically enhanced sensitivity to anticancer drugs in CD133-positive HCC but not CD133-negative HCC (Fig. 5d).

To confirm our results, we further investigated whether pretreatment with sulfasalazine affected drug resistance on LCSCs spheroids. Combination treatment with SASP and existing anticancer therapies such as cisplatin, doxorubicin significantly attenuated the strong drug resistance of LCSCs spheroids (Fig. 5e). We also investigated the effects of combination treatment with SASP and anticancer therapies in primary HCC tumors to provide better physiological relevance and applicability, and found pronounced therapeutic efficacy when SASP was combined with cisplatin and doxorubicin to treat 4 kinds of human primary HCC cell types (Fig. 5f).

**Fig. 6:**
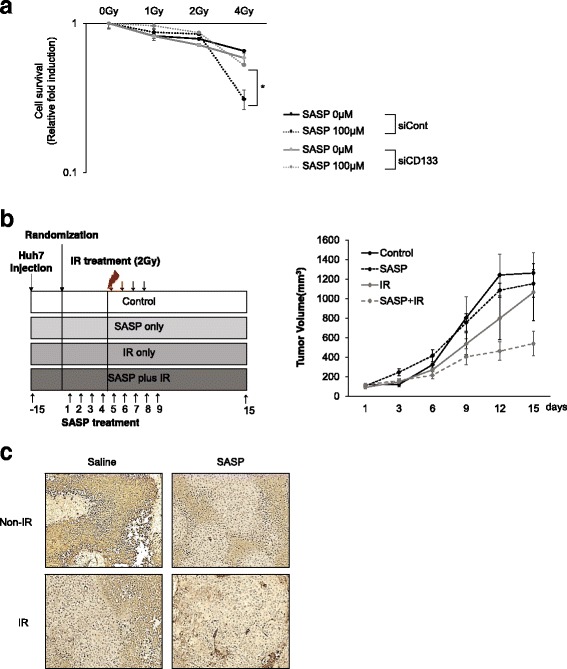
Pretreatment with SASP eliminates the CD133-positive HCC population combined with anticancer therapies in vivo and in vitro*.* **a** The colony-forming assay was performed in Huh7-siCont and Huh7-siCD133 cells after treatment with 1, 2, and 4 Gy of radiation and pretreatment with 100 μM SASP. After 10 days, colonies were stained with tryphan blue and counted. **b** Schematic of experimental procedure in vivo. When the tumor volume reached 200–250 mm^3^, mice were randomized to 4 groups (Control, SASP, IR (2Gy), SASP plus IR), and 250 mg/kg sulfasalazine were injected for 9 days. During the sulfasalazine injection, mice were irradiated with 2Gy dose for 4 days (*left panel*). The volume of the tumor was examined every 2 days in the indicated groups. Data are mean ± SD for three animals in each group (*right panel*). **c** Immunostaining of CD133 in Huh7 tumors of indicated groups in xenografted mice

Ionizing radiation (IR) instantaneously causes the formation of water radiolysis products that contain some ROS. We also investigated whether pretreatment with sulfasalazine induced synergistic effect with IR treatment in HCC. A colony-forming assay revealed that pretreatment with SASP could enhance the efficiency of IR therapy in HCC with high levels of CD133 (Fig. 6a).

To determine whether SASP could sensitize an in vivo system to IR, we examined the growth of implanted Huh7 xenograft tumors. Administration of SASP alone showed just a subtle reduction of tumor growth and IR alone did not induced significant tumor regression. However, IR plus pretreatment with SASP dramatically inhibited tumor volume, versus mice treated with IR alone (Fig. 6b). These data suggested that SASP could be sensitizer for highly efficient treatment of IR.

Next, we examined whether SASP has a sensitizing effect on CD133-positive cells, specifically in HCC. Immunohistochemical analysis revealed that IR plus pretreatment with SASP significantly reduced the CD133-positive cell population in Huh7 tumor cells in vivo compared with treatment with IR alone (Fig. 6c). These results indicate that SASP can sensitize CD133-positive HCC cells to available anti-cancer therapies by reducing their ROS defense capacity. Taken together, our data suggest that treatment with SASP suppresses GSH synthesis and increases ROS levels in CD133-positive HCC cells, thereby facilitating the robust therapeutic activity of combined SASP and anticancer therapies in human liver carcinomas.

## Discussion

HCC is one of the most malignant human cancers, with high mortality rates worldwide in spite of early detection and improvements in therapeutic technology. Nowadays, surgical resection is considered as a first-line therapy for HCC, whereas systemic chemotherapy plays an integral role for patients with advanced HCC for whom surgery is not a feasible option [29]. However, the effects of chemotherapeutics such as sorafenib [30] and cisplatin [31] on advanced HCC are extremely limited, because most types of HCC inherently possess drug resistance to chemotherapy [32]. CSCs are considered the ‘Achilles heel’ of anticancer efforts, due to their strong resistance to chemotherapy and radiotherapy. Recent studies indicate that HCC progression and drug resistance might be derived from CSCs [33]. Papers have shown that CSC-related surface markers and pathways can modulate tumor development and suppression in liver cancer [9, 34–36]. Although the existence of CSCs in solid human tumors is widely accepted, details of their origin and the source of their chemoresistance are unclear [37]. In reality, the culture and functional study of CSCs are difficult in vitro, because CSC enrichment is rapidly lost in artificial culture systems [38]. In order to overcome this, we applied an alternative approach to enrich the CSC population by manipulating 3D culture conditions. From these attempts, we discovered that characteristics and population of CSCs are controlled by changes in the tumor microenvironment, and that CD133-positive HCC cells have CSC-like properties to maintain tumor survival from anti-cancer therapies (Fig. 1). CD133/Prominin-1 has attracted considerable attention as a representative liver CSC marker. Indeed, liver cancer patients with high CD133 expression levels were found to have shorter overall survival and higher recurrence rates than patients with low CD133 expression (13). Studies have shown that CD133-positive liver CSCs can induce aberrant signaling pathways different from those in CD133-negative cells, such as the Akt/PKB pathway, JNK, mTOR, ERK, and β-catenin *etc.* [9, 39, 40]. However, the specific mechanism of action by which CD133 CSCs are able to avoid conventional therapies in HCC remains unknown. Here, we revealed that HCC cells with high CD133 expression levels have a strong capacity for ROS defense compared to HCC cells with low levels of CD133 expression (Figs. 2, 3 and 4). Although the mechanisms regulating the expression of CD133 in hypoxic conditions are known [41], the detailed mechanism by which CD133 expression is upregulated in response to oxidative stress has not been elucidated. Here, our data showed that CD133 expression is increased in HCC in response to oxidative stress (Fig. 2e, f).

Recently, the correlation between ROS status and chemo- and radio-resistance in CSCs has been revealed in diverse cancers. A subset of CSCs exhibits enhanced ROS defense compared to non-tumorigenic cells in breast tumors [19] and lower intracellular concentrations of ROS and ATP can be used as indicators of CSCs in lung cancer [20]. However, the mechanisms by which CSCs maintain lower levels of ROS in HCC remain hitherto unknown.

In the present study, we have found that CD133-positive HCC cells control intracellular ROS level via the upregulating of GSH and sulfasalazine (SASP) not only alleviates ROS defense capacity but also increases the therapeutic efficacy of conventional anticancer therapy in CD133-positive HCC cells but not in CD133-negative HCC cells in vivo and in vitro.

Actually, this kind of mechanism was proposed by Ishimoto et al. for another stem-like protein, CD44 [22]. They demonstrated that ablation of CD44 induced loss of xCT from the cell surface and suppressed tumor growth in gastric cancer. Here, we examined a potential cross-talk between CD133 and CD44 on ROS status (Fig. 3b) and we found that CD133-positive cells perform defense against ROS with proposed mechanism which is indifferent of CD44 expression in HCC.

In this study, we pre-treated sulfasalazine (SASP) before treating anti-cancer drugs or radiation. We would like to emphasize the SASP with sensitizer for increasing the drug efficiency through increasing the ROS accumulation and decreasing the GSH. Additionally, we could hypothesis that CD133 inhibits ROS resistance through the maintenance of ROS-induced increasing xCT expression in CD133-positive HCC cells, and thereby plays a central role in resistance to liver cancer therapy. xCT inhibition by treatment with SASP could sensitize CD133-positive HCC cells to available anticancer therapies.

SASP not only alleviates ROS defense capacity but also increases the therapeutic efficacy of conventional anticancer therapy in CD133-positive HCC cells but not in CD133-negative HCC cells in vivo and in vitro (Figs. 5 and 6). To date, no single agent or combination therapy has demonstrated any advantage in terms of both overall survival and quality of life, representing an unmet need. Combination therapy has not improved overall survival but has nonetheless been in wide use for many years because of its possible roles in palliation. Thus, we herein suggest that combination therapy with SASP and existing anticancer therapies should be feasible for patients with HCC without imposing side effects, since SASP is already approved to treat rheumatoid arthritis without safety issues. Given that CD133-positive HCC cells play a central role in resistance to cancer therapy, we believe that selective inhibition of the CD133-positive HCC population by pretreatment with SASP should surpass the limitations of the existing treatment of liver cancer.

## Conclusions

In the conclusion, our results provide clear evidence that CD133 elevates ROS resistance through enhancing of glutathione (GSH) levels, and thereby plays a central role in resistance to liver cancer therapy. GHS inhibition by treatment with SASP sensitizes CD133-positive HCC to available anticancer therapies. Therefore, our results suggest that a combination of sulfasalazine and conventional chemotherapy might be a promising approach to overcoming resistance to therapy in liver cancer.

## Additional file

Additional file 1: Figure S1. Liver cancer stem cell (LCSC) spheroids express a lower level of hepatocyte-related mRNA and higher level of stem cell-related mRNA compared to hepatocellular carcinoma cell (HCC) spheroids. **Figure S2**. LCSC spheroids have strong resistance to anti-cancer drugs compared to HCC spheroid. **Figure S3**. Knockdown of CD133 (Depletion of CD133) does not affect the antioxidant gene in HCC. **Figure S4**. Sulfasalazine (SASP) inhibits activity of GSH in CD133-positive cells specifically. (DOCX 366 kb)
