# Supplementary material for: Sulfasalazine attenuates evading anticancer response of CD133-positive hepatocellular carcinoma cells
Source: J Exp Clin Cancer Res. 2017 Mar 3;36:38. doi: 10.1186/s13046-017-0511-7 (PMC5335796; doi:10.1186/s13046-017-0511-7)
Supplement: Additional file 1: Figure S1. — Liver cancer stem cell (LCSC) spheroids express a lower level of hepatocyte-related mRNA and higher level of stem cell-related mRNA compared to hepatocellular carcinoma cell (HCC) spheroids. Figure S2. LCSC spheroids have strong resistance to anti-cancer drugs compared to HCC spheroid. Figure S3. Knockdown of CD133 (Depletion of CD133) does not affect the antioxidant gene in HCC. Figure S4. Sulfasalazine (SASP) inhibits activity of GSH in CD133-positive cells specifically. (DOCX 366 kb) [file 13046_2017_511_MOESM1_ESM.docx]

**Sulfasalazine attenuates** **evading anticancer response of CD133-positive hepatocellular carcinoma cells**

**SUPPLEMENTARY FIGURE AND TABLE**

**
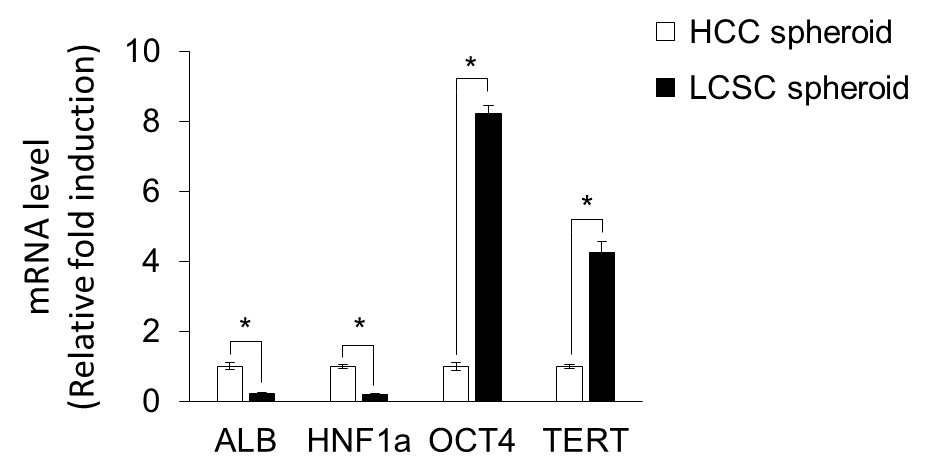
**

**Supplementary Figure 1. Liver cancer stem cell (LCSC) spheroids express a lower level of hepatocyte-related mRNA and higher level of stem cell-related mRNA compared to hepatocellular carcinoma cell (HCC) spheroids.** Expression of hepatocyte-related genes (ALB, HNF1a) and stem cell-related genes (OCT4, TERT) in HCC spheroids and LCSC spheroids using the realtime PCR. Values were normalized to GAPDH. Data are shown as means ± SD from two independent experiments with duplicates. *P<0.05.


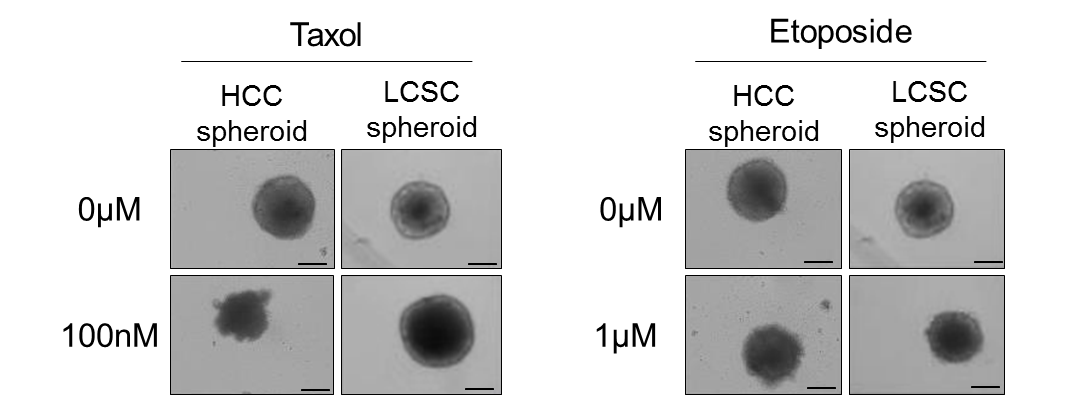


**Supplementary Figure 2. LCSC spheroids have strong resistance to anti-cancer drugs compared to HCC spheroid.** LCSC spheroid and HCC spheroid were treated with 100nM of Taxol (left) and 1µM of Etoposide (right). Images were taken on day 8 of drug treatment. Scale bar = 200µm.

**
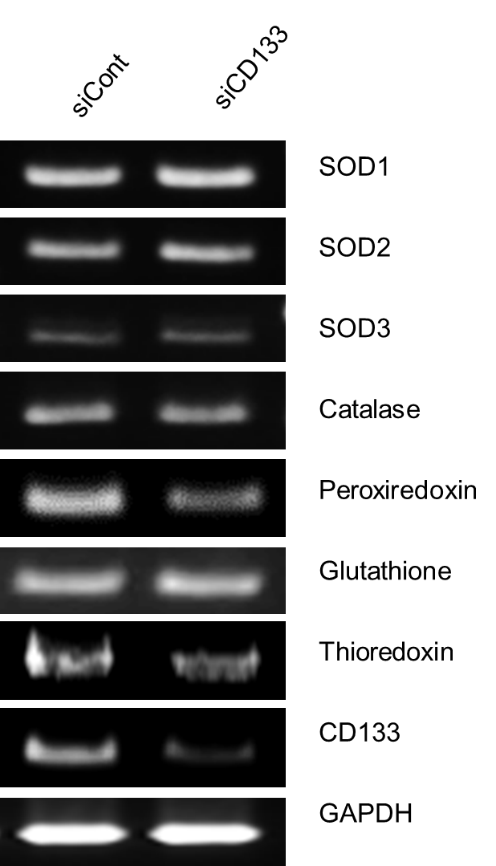
**

**Supplementary Figure 3.** **Knockdown of CD133 (Depletion of CD133) does not affect the antioxidant gene in HCC**. Expression of the indicated antioxidant enzyme genes in Huh7 cells transfected with siRNA (siCont, siCD133). Total RNA isolated from the Huh7-siCont and Huh7-siCD133 cells was subjected to RT-PCR analysis with primers related to antioxidant enzymes. Expression of the gene for GAPDH was examined as a control.

**
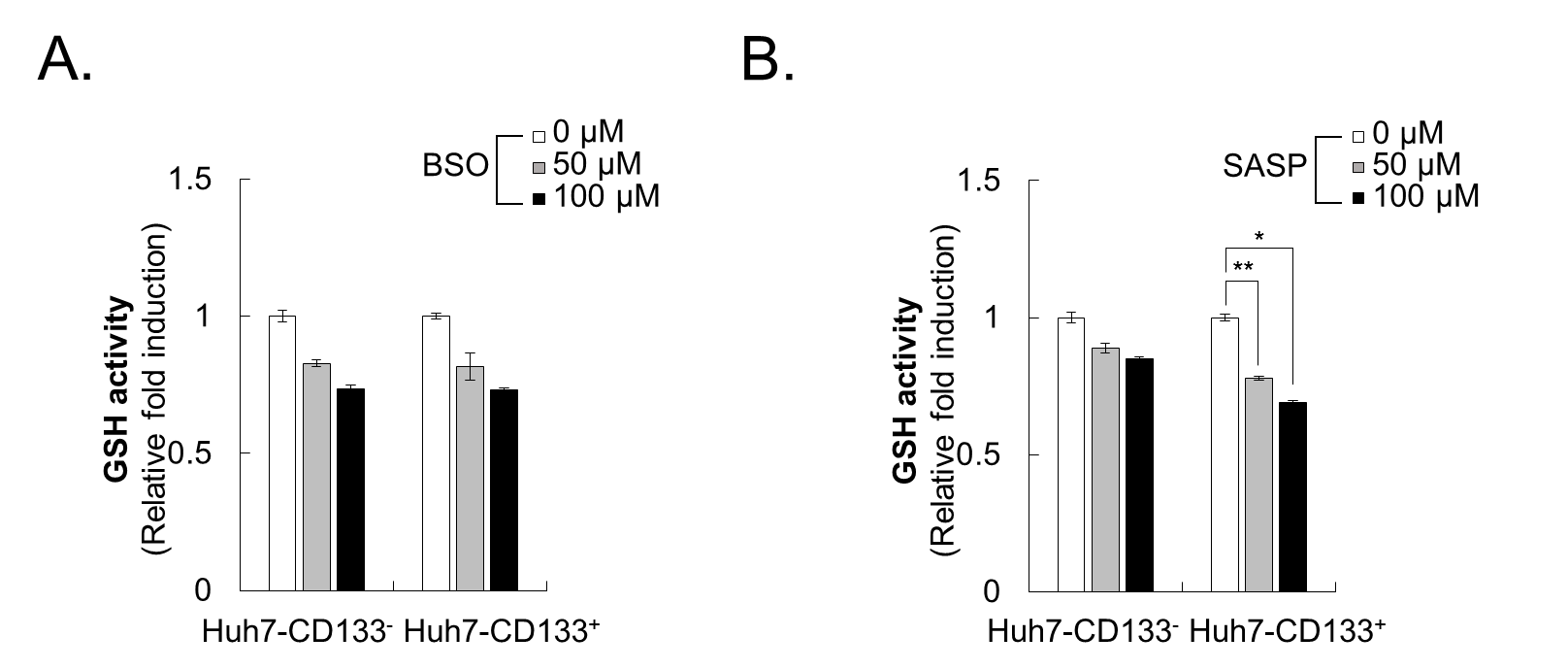
**

**Supplementary Figure 4. Sulfasalazine (SASP) inhibits activity of GSH in CD133-positive cells specifically.** GSH activity was measured by treating with the indicated concentration of (A) guthionine sulphoximine (BSO), and (B) SASP for 24hr in CD133-negative and CD133-positive HCC cells. All data are mean values ± SD from two independent experiments. *p<0.05, **p<0.005.
